# Expression of Protein Acetylation Regulators During Peripheral Nerve Development, Injury, and Regeneration

**DOI:** 10.3389/fnmol.2022.888523

**Published:** 2022-05-17

**Authors:** Junjie Sun, Yuhua Ji, Qingyun Liang, Mengru Ming, Yuhan Chen, Qi Zhang, Songlin Zhou, Mi Shen, Fei Ding

**Affiliations:** Key Laboratory of Neuroregeneration of Jiangsu and Ministry of Education, NMPA Key Laboratory for Research and Evaluation of Tissue Engineering Technology Products, Jiangsu Clinical Medicine Center of Tissue Engineering and Nerve Injury Repair, Co-Innovation Center of Neuroregeneration, Nantong University, Nantong, China

**Keywords:** sciatic nerve, protein acetylation, HDAC, development, regeneration

## Abstract

Protein acetylation, regulated by acetyltransferases and deacetylases, is an important post-translational modification that is involved in numerous physiological and pathological changes in peripheral nerves. There is still no systematical analysis on the expression changes of protein acetylation regulators during sciatic nerve development, injury, and regeneration. Here, we sequenced and analyzed the transcriptome of mouse sciatic nerves during development and after injury. We found that the changes in the expression of most regulators followed the rule that “development is consistent with regeneration and opposite to injury.” Immunoblotting with pan-acetylated antibodies also revealed that development and regeneration are a process of increased acetylation, while injury is a process of decreased acetylation. Moreover, we used bioinformatics methods to analyze the possible downstream molecules of two key regulators, histone deacetylase 1 (Hdac1) and lysine acetyltransferase 2b (Kat2b), and found that they were associated with many genes that regulate the cell cycle. Our findings provide an insight into the association of sciatic nerve development, injury, and regeneration from the perspective of protein acetylation.

## Introduction

The peripheral nervous system (PNS) is a stable structure with nerve axons and myelin sheathes surrounding the axons and mainly functions to connect peripheral tissues and organs to the nerve system. Schwann cells (SCs) is the myelinating cells in the PNS, accounting for the highest proportion of cell types in peripheral nerves. Due to the great plasticity of SCs, the PNS exhibits a higher regenerative capacity than the central nervous system (CNS) (Nocera and Jacob, 2020). Numerous studies have shown that after peripheral nerve injury, nerve regeneration is a developmental reappearance that is morphologically characterized by myelination and lamellar thickening. In addition, at the molecular level, it is accompanied by the activation of Hdac1/2, SOX10, and NF-κB (Salzer, 2015; Nocera and Jacob, 2020).

Hdac1/2 is an important regulator of protein acetylation. Acetylation refers to the transfer of acetyl groups into lysine residues, which is one of the major post-translational modifications in cells. Protein acetylation can be grouped into two types. Histone acetylation as the first type determines the assembly of histones and the tightness of their interaction with DNA. Therefore, changes in histone acetylation can theoretically regulate the transcription of various genes. The second type is the acetylation of non-histone proteins that are mostly distributed in cytoskeleton proteins, which is related to physiological activities such as cell morphology, intracellular transport, cell division, and movement (Verdin and Ott, 2015). During development, protein acetylation is under strict control. Mice with Hdac1 deficiency exhibited proliferation defects and growth retardation and died before embryonic development day 10 (Lagger et al., 2002; Montgomery et al., 2007). Hdac1 is found to regulate specific gene programs by activating or repressing certain promoters in embryonic cells. Histones H3 and H4 are hyperacetylated and cyclin-dependent kinase inhibitors p21 and p27 are upregulated in Hdac1-deficient embryonic stem cells (Zupkovitz et al., 2006). Acetylation is also widely observed in peripheral nerves and many important proteins related to myelination, such as Periaxin (PRX), 2′,3′-cyclic nucleotide-3′-phosphodiesterase (CNPase), and c-jun, have been confirmed to function *via* relevant acetylation modifications (Parmigiani et al., 2008; Zhang et al., 2019; Wang et al., 2021a).

Intracellular acetylation is a reversible dynamic equilibrium process that is co-regulated by acetyltransferases and deacetylases. There are 13 known human and mouse lysine acetyltransferases, including Kat1 (also known as Hat1), Kat2A (also known as GCN5), Kat2B (also known as PCAF), Kat3A (also known as Crebbp), Kat3B (also known as Ep300), Kat5 (also known as Tip60), Kat6A (also known as MOZ), Kat6B (also known as MORF), Kat7 (also known as HBO1), Kat8 (also known as MOF), α-tubulin N-acetyltransferase 1 (Atat1), establishment of cohesion 1 homolog 1 (Esco1) and Esco2. According to different products, HDAC family members belong to two groups, Zn2+-dependent classical deacetylases family (Hdac1–11) and NAD+-dependent sirtuin deacetylases family (Sirt1–7). In addition, lymphoid enhancer Binding factor 1 (Lef1) and T cell-specific transcription factor 1 (TCF1; also known as TCF7) have also recently been reported to have deacetylated functions (Narita et al., 2019).

Some acetylation regulators have widespread effects on myelin development and regeneration of peripheral nerves. Histone deacetylase 1 (Hdac1) and Hdac2 were the first to be studied. Mice with Hdac1/2 deficiency exhibited severe myelin deficiency with SCs developmentally arrested at the immature stage (Chen et al., 2011). When the sciatic nerve, a common kind of peripheral nerve, is injured, an early intervention with Hdac1/2 inhibitors can promote remyelination and enhance functional recovery (Brugger et al., 2017). In addition, Hdac3, 4, and 5 are all involved in the development and regeneration of the myelin sheath in peripheral nerves (Gomis-Coloma et al., 2018; He et al., 2018). Another study indicated that the acetylation level of eEF1A1 is determined by the disruption of Tip60/Hdac2 balance, thereby regulating the activation of Sox10 target genes and then controlling remyelination efficiency in the PNS and CNS (Duman et al., 2020). So, the coordination of various regulators triggers protein acetylation in the sciatic nerve. However, there is no relevant report on the expression patterns of protein acetylation regulators during sciatic nerve development, injury, and regeneration.

Here, we constructed a model of sciatic nerve development and injury and systematically analyzed the expression changes of protein acetylation regulators using RNA-seq. We also confirmed using Western blot assay that the acetylation level of the sciatic nerve increases during development and regeneration and decreases during injury. All these changes may be under the control of deacetylases Hdac1/2 and acetylases Kat2b and Esco1. Finally, we found that Hdac1 and Kat2b share numerous downstream molecules, most of which could be clustered into pathways related to transcriptional regulation and cell cycle regulation. Our data suggest an intriguing mechanism underlying neural development, injury, and regeneration, and emphasize that acetylation is an important mediator.

## Materials and Methods

### Experimental Animals

C57BL/6 mice (10 weeks, male) used in this study were provided by the Experimental Animal Center of Nantong University. All experimental animals were housed in a specific pathogen-free barrier system under a 12-hour light/dark cycle at 23 ± 2°C and 60% humidity. All animal experiments were approved by the Animal Ethics Committee of Nantong University and followed the International guidelines (NIH) for the Care and Use of Laboratory Animals. The tester was qualified to have the Jiangsu Provincial Laboratory Animal Practitioner Post Certificate with a certificate No. 220212305.

### Animal Modeling

Mice were caged together. The emergence of a vaginal plug indicates the appropriate time for mating. Fetuses of embryonic day 20 were identified. Depending on their birth time, 1-, 4-, 7-, 14-, 21-day, 1-, 3-, 6-, and 12-month-old mice were identified. The sciatic nerve crush injury in C57BL/6 mice was induced by applying constant pressure to the nerve with a No. 5 jeweler’s forceps for 15 s. Injured sites were marked using carbon power, and the marker center was used to distinguish proximal and distal nerve stumps.

### RNA Extraction

Total RNA samples were extracted from fresh or frozen tissues using the well-established TRIzol method. Briefly, TRIzol (Cat#15596018, Invitrogen) was used to immerse tissue samples, and a tissue crusher was used to completely dissolve these samples. Chloroform and isopropanol were sequentially added to aid RNA extraction and crystallization, respectively. The RNA pellet was rinsed twice with 75% ethanol. Concentration and OD260/OD280 were determined using a NanoDrop 2000 spectrophotometer (Thermo Scientific, United States), and agarose gel electrophoresis was used to detect RNA integrity to ensure RNA quality for subsequent sequencing or qPCR.

### Library Construction, Sequencing, and Data Preprocessing

The transcriptome sequencing and analysis were conducted by OE Biotech Co., Ltd. (Shanghai, China). RNA samples from mouse sciatic nerves were processed according to the manufacturer’s instructions (TruSeq^®^ Stranded mRNA Sample Preparation Guide, Part # 15031047 Rev. D. September 2012) to build the library. Briefly, 4 μg of DNase-treated total RNA was incubated with magnetic beads with Oligo (dT) to capture the mRNAs with poly-A tails. The mRNAs were broken into short fragments by addition of fragmentation buffer. With the fragmented mRNA as a template and random hexamers primers, the first strand of cDNA was synthesized. The second-strand cDNA was synthesized in the prepared second-strand synthesis system. The double-strand cDNA was purified using a kit. The purified double-strand cDNA was then subjected to end repair, A-tailing, and adapter ligation for final PCR amplification. The constructed library was quality-checked with an Agilent 2100 Bioanalyzer (Agilent Technologies, Santa Clara, CA, United States), and then sequenced using the Illumina HiSeq X Ten platform (Illumina, San Diego, CA, United States) to generate double-ended data of 125 or 150 bp.

Hisat2 (Kim et al., 2015) was used to align the Raw Reads with the specified reference genome, to obtain the position information of the reads on the genome. The number of reads matched to protein-coding genes in each sample was counted using the htseq-count software (Anders et al., 2015), and converted into protein-coding gene expression levels and FPKM values according to the formula. The DESeq (Love et al., 2014) software was used to standardize the number of counts for each gene in each sample and the expression level of each gene was estimated using the BaseMean value. Fold of difference was calculated. Negative binomial distribution test was adopted to test the significance of the difference in the number of reads. Finally, the differential expression fold and the difference significance test results (*P*-value) were obtained.

### Reverse Transcription Quantitative Polymerase Chain Reaction

DNA removal and cDNA synthesis were performed using HiScript II Q RT SuperMix for qPCR (+gDNA wiper) kit (Cat#R223-01, Vazyme, China). Reactions were as follows: first total RNA (1 μg), 4 × *g* DNA Wiper Mix (4 μL), and DEPC H2O were mixed into 16 μL, at 42°C for 2 min to remove DNA; 4 μL of 5 × HiScript II qRT SuperMix II was added at 50°C for 1 min and then at 85°C for 5 s to synthesize cDNA. Quantitative PCR reactions were performed on cDNA using ChamQ Universal SYBR qPCR Master Mix (Cat#Q711-02, Vazyme, China) in StepOnePlus*™* Real-Time PCR System sequence detection system (Thermo Fisher Scientific). The 2^–ΔΔCt^ method was used to calculate relative changes of gene expression. Actb was used as the reference gene for PCR normalization. The primers are as follows:

**Table:** 

No.	Gene symbol	Forward (5′–3′)	Reverse (5′–3′)
1	Hdac1	GTGAACTACCCACTGCGA	TAGGCTGGAACATCTCCATTAC
2	Hdac2	CATCAGACAAACGGATAGCTT	ATCAGCAACATTCCTACGAC
3	Kat2b	CCATTTGAGAAGCCCAGTATT	TGTCTGCCTCTCTTTCGAT
4	Esco2	AAGACCAGCTCGTCATTG	TTCAGGGTTGGAAGCAGTATAG
5	Sirt2	CAGTTCAAGCCAACCATCT	CTCGTTCCAGCGTGTCTA
6	Hdac11	ATGTTTACAACCGCCACATCTA	CTCTCCACCTTCTCCAGATATT
7	Actb	GGCTCCTAGCACCATGAAGA	AGCTCAGTAACAGTCCGCC

### Modular Definition of Differentially Expressed Genes

Considering gene expression changes during development, injury, and regeneration, a *P*-value less than 0.05 indicates no differential expression and a *P*-value higher than 0.05 indicates differential expression. Three modules described in Figure 3 were defined based on the rule that “the change trend of target gene expression during development is consistent with that during regeneration but opposite to that during injury.” There are two possibilities for a gene to be judged as module 1: the first is that all three processes follow the above rule, and the second is that two processes meet the above rule with the third process unchanged. Genes with expression changes that do not conform to the above rule in ≥2 processes are judged as module 2. Genes with no expression changes in ≥2 processes were classified as module 3. Therefore, module 1 indicates basically consistent, module 2 indicates discrepancy, and module 3 indicates no change.

### Western Blot Assay

Fresh tissue samples were immersed in protein lysis buffer containing protease inhibitors and crushed using a crusher until completely dissolved. Lysate supernatant was mixed with loading buffer at a ratio of 4:1, and the mixture was boiled at 95°C for 10 min to obtain whole protein supernatant. Protein samples were quantified by spectrometer to ensure a total protein of 20 μg per lane. Protein samples separated by SDS-PAGE gel electrophoresis were transferred to wetted PVDF membranes (Millipore, Bedford, MA, United States) using the common Western blot method. To measure the amount of acetylated tissue, immunoblotting assay with anti-acetyllysine mouse mAb (Cat# PTM-101, PTM BIO, China) as primary antibody and Goat Anti-Mouse IgG H&L (Cat# ab205719, Abcam, United States) as secondary antibody was performed. The immunoblots were visualized using the enhanced chemiluminescence kit (Beyotime, Shanghai, China). Immunoblot intensities were quantified by ImageJ software.^1^ The total gray value of all detectable bands in each lane was used for pan-acetylation quantification in Figure 4. Ponceau S staining was used to visualize total protein across the membrane.

### Prediction of Interactive Molecules

Two databases were used to analyze the potential regulatory networks of Hdac1 and Kat2b. RRUST database (Han et al., 2018) was used to analyze the interaction of Hdac1 and Kat2b as transcription factors with the target, and the Gene Ontology biological process terms of the target were also provided by this database. The protein–protein interactions and interaction scores of Hdac1 and Kat2b with the target were downloaded from the STRING database (Szklarczyk et al., 2017). For interaction scores, Score > 200 was taken as cut-off. Venny was used to identify overlaps between downstream molecules (also included in Figure 5). DAVID (Huang et al., 2009) was used to analyze the Gene Ontology biological process terms of overlapping molecules. With target as node and score as edge, Cytoscape software^2^ was used to generate interaction diagrams.

### Statistical Analysis

All statistical analyses were performed using SPSS 20.0 (IBM, United States) and experimental data were presented as SD ± SEM. SPSS Pearson analysis was used to calculate *R* and *P*-values. Intergroup comparison was done using one-way analysis of variance and *P*-values were thus calculated.

## Results

### Expression Changes of Acetyltransferase and Deacetylase During Sciatic Nerve Development and Injury

We prepared relevant animal models and conducted deep sequencing to systematically analyze the expression changes of acetyltransferases and deacetylases during sciatic nerve development and regeneration. For the developmental process, sampling was done at intensive time points, including embryonic day 20, postnatal days 1, 7, 14, and 21, postnatal months 1, 3, 6, 9, and 12, throughout the embryonic, postnatal, juvenile, and adult stages. For the regeneration process, we prepared the animal model of sciatic nerve clamping and set five observation time points, including postoperative days 1, 3, 7, 14, and 21, for proximal (proximal nerve stump, p) and distal (distal nerve stump, d) sampling according to the location of the injury. Total RNA was extracted to generate RNA-seq data. Each sample contained 40–50 million raw reads, and the Q30 value of all samples was >91.4%, suggesting that the sequencing depth and data are sufficiently available for subsequent analysis. The expression of all detected genes in developmental and injury samples is listed in Supplementary Files 1, 2.

**FIGURE 1:**
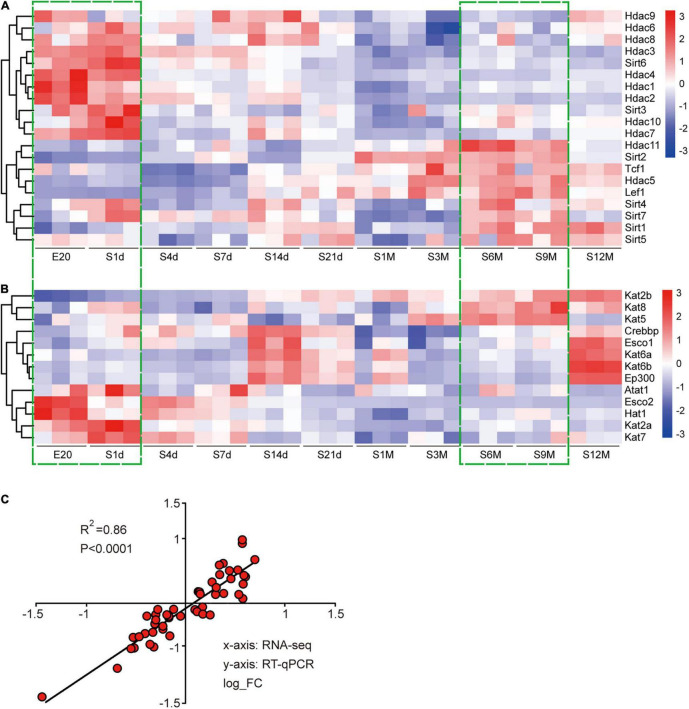
Expression analysis of acetyltransferases and deacetylases during sciatic nerve development. **(A)** Heat map of the expression of 20 deacetylases during sciatic nerve development. **(B)** Heat map of the expression of 13 acetyltransferases during sciatic nerve development. Genes with similar expression characteristics are grouped into category. The FPKM value of gene expression is converted into fold. The darker red indicates the greater fold for up-regulation; and the darker blue indicates the greater fold for down-regulation. The two time periods with the most drastic changes in gene expression are marked by green dashed boxes (E20 and S1d vs. S6m and S9m). **(C)** Correlation analysis of RT-qPCR and sequencing results. The *X*-axis represents RNA-seq results and the *Y*-axis represents RT-qPCR results. Differential expression folds are log-transformed, and *P*-values are calculated by Pearson correction coefficients.

In this study, we identified the coding genes of 20 deacetylases and 13 acetyltransferases and analyzed their expression changes using the Cluster heatmap. During development, deacetylases could be clearly divided into two categories, one containing most of the Hdac family members that were gradually down-regulated and the other containing most of the Sirt family members that were gradually up-regulated (Figure 1A). There was no uniform expression characteristics of acetyltransferases. The expression of Kat2b/5/8 was gradually up-regulated, the expression of Atat1, Esco2, Hat2, Kat2a, and Kat7 was gradually down-regulated, and the expression of other genes first increased and then decreased (Figure 1B). Overall, almost all genes expressed very similarly at E20 and 1d, and then their expression changed in a constant state until the greatest amount of change was achieved and stabilized at 6–9 months after birth (Figures 1A,B). To ensure the reliability of the sequencing results, we randomly selected five of the above genes to perform reverse transcription quantitative polymerase chain reaction (RT-qPCR) assay. Correlation coefficients were used for evaluating the consistency between experimental and sequencing results, and a value of *R*^2^ = 0.86 indicated a high correlation between the two (Figure 1C).

**FIGURE 2:**
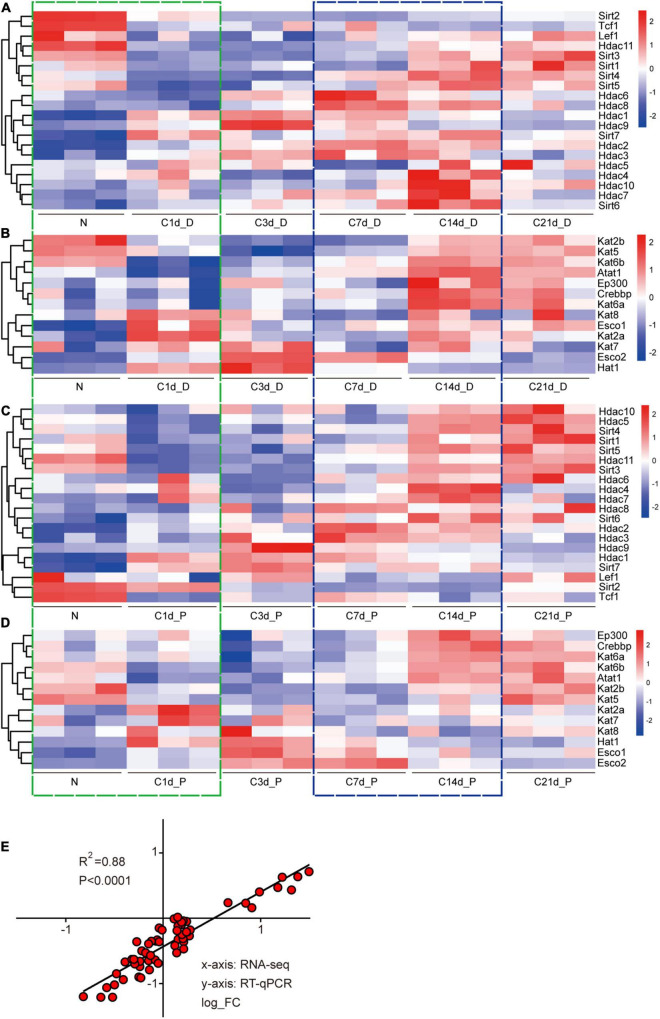
Expression analysis of acetyltransferases and deacetylases after sciatic nerve injury. Heat map of the expression of 20 deacetylases during sciatic nerve injury (**A:** distal nerve stump; **C:** proximal nerve stump). Heat map of the expression of 13 acetyltransferases during sciatic nerve injury (**B:** distal nerve stump; **D:** proximal nerve stump). Genes with similar expression characteristics are grouped into one category. The FPKM value of gene expression is converted into fold. The darker red indicates the greater fold value for up-regulation; and the darker blue indicates the greater fold value for down-regulation. The two time periods with the most drastic changes in gene expression are marked by green (N vs. C1d) and blue (C7d vs. C14d) dashed boxes. **(E)** Correlation analysis of RT-qPCR and sequencing results. The *X*-axis represents RNA-seq results and the *Y*-axis represents RT-qPCR results. Differential expression folds are log2 transformed, and *P*-values are calculated by Pearson correction coefficients.

Expression of acetyltransferases and deacetylases showed a complex variation with no discerning trend during sciatic nerve injury and regeneration. Unsurprisingly, the gene expression patterns of proximal and distal nerve stumps were not identical, which is consistent with the different physiological changes of proximal and distal nerve stumps (Min et al., 2021). However, there were clear and noticeable gene expression transitions for both acetylases and deacetylases at two periods, N-1d (Figures 2A–D; green boxes) and C7d-C14d (Figures 2A–D; blue boxes), corresponding to the injury period and regeneration periods. This implicates that acetylation shows more dramatic changes at these two periods than the other time periods. Interestingly, during the injury time period (N-1d) of the distal nerve stump samples, the expression pattern of Sirt genes is as follows: most of the Sirt family members were down-regulated, while most of the Hdac family were up-regulated (Figure 2A). This trend, which is opposite to that during development (Figures 1A vs. 2A), suggests a possible link between the expression of acetylated regulators during development, injury, and regeneration. We performed qPCR to verify this part of the sequencing results, and the two also showed high correlation (Figure 2E).

**FIGURE 3:**
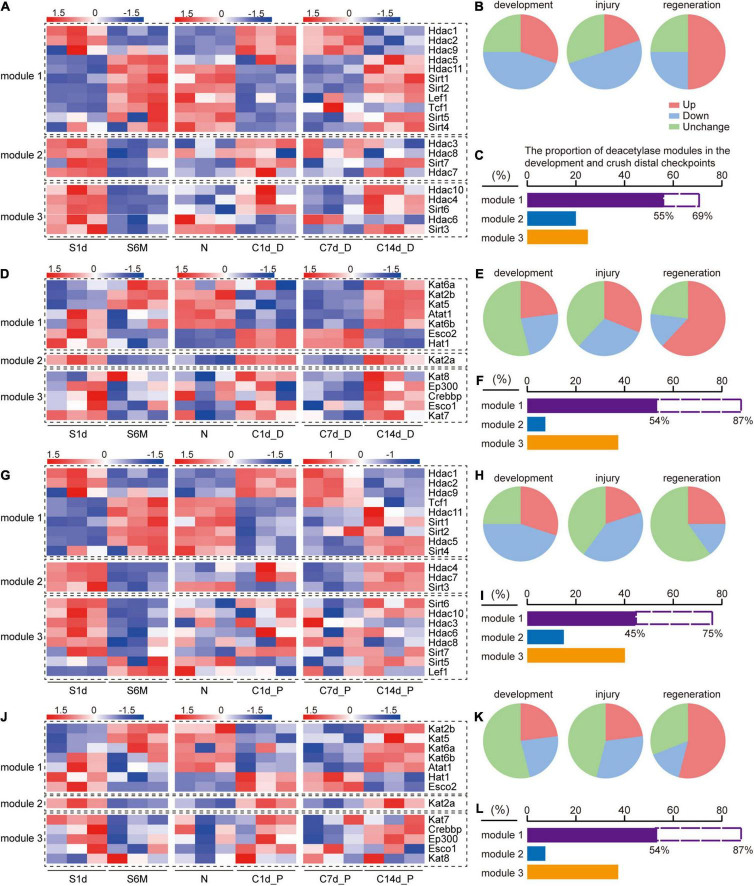
Comparison of expression trends of acetylation regulators during development, injury, and regeneration. S1d-S6m represents the developmental process, N-C1d represents the injury process, and C7d-C14d represents the regeneration process. Modular classification of acetylation regulators is done according to “whether this gene expression trend is the same during development and regeneration and opposite to that during injury.” **(A)** Deacetylase, distal nerve stump; **(D)** acetyltransferase, distal nerve stump; **(G)** deacetylase, proximal nerve stump; **(J)** acetyltransferase, proximal nerve stump. Each module is separated by a black dashed box. Panels **(B,C)** describe the information on the statistics and interpretations of panel **(A)**. **(B)** Three pie charts correspond to development, injury, and regeneration stages in panel **(A)**, respectively. Red, green, and blue color in each pie chart represent the proportions of genes that are up-regulated, down-regulated, and have no expression change, respectively. Similarly, panels **(E,H,K)** correspond to panels **(D,G,J)**, respectively. **(C)** The proportion of the number of genes in each module of panel **(A)**. The purple column represents the proportion of module 1 in the three modules, and the dashed blank column represents the proportion of module 1 in module 1 and module 2. Similarly, panels **(F,I,L)**. correspond to panels **(D,G,J)**, respectively.

**FIGURE 4:**
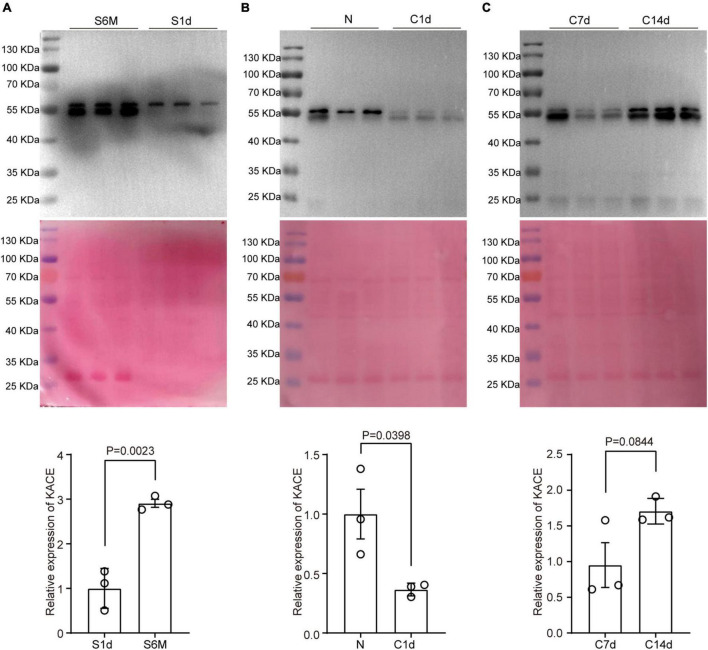
Changes in protein pan-acetylation during sciatic nerve development, injury, and regeneration. Expression analysis of pan-acetylation during development **(A)**, injury **(B)**, and regeneration **(C)**. The upper panel refers to Western blot results. The middle panel refers to Coomassie brilliant blue staining results that reflect the total amount of proteins on the PVDF membrane. The lower panel refers to statistical results. Bars represent the mean ± SEM, and the *P*-value is calculated by Student’s *T*-test, *n* = 3.

**FIGURE 5:**
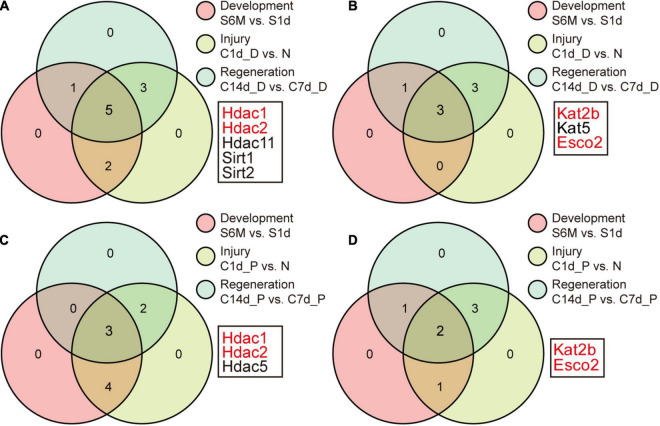
Identification of key acetylation regulators. **(A)** Identification of the overlap genes in module 1 of Figure 3A. Genes with no change in each process are removed and the remaining genes in the three processes are intersected. Panels **(B–D)** correspond to Figure 3D,G,J, respectively. The selected genes (marked in red) represent the differentially expressed genes in module 1 in any process, indicating that the genes may mediate acetylation changes during development, injury, and regeneration.

### Expression Characteristics of Acetyltransferases and Deacetylases During the Development, Injury, and Regeneration of Sciatic Nerves

Based on the above clues, we hypothesized that there are common rules for the changes in the expression of acetylated regulators during sciatic nerve development, injury, and regeneration. We independently defined three gene expression modules for sorting acetyltransferase and deacetylase encoding genes during three time periods: 1d-6m (development), N-C1d (injury), and C7d-C14d (regeneration). According to the rule that “the target gene shows the same changes in the development and regeneration process but an opposite trend in the injury process,” module 1 indicates the expression of the target gene basically follows this rule, module 2 indicates the expression of the target gene does not follow this rule, and module 3 indicates no expression change (more details in section “Materials and Methods”). Modular classifications were conducted four times according to the type of each enzyme and the location of each sample. Genes and their expression changes contained in each module were displayed on a heat map (Figures 3A,D,G,J). The proportion of differentially and non-differentially expressed genes was used to assess whether acetylation and deacetylation were activated during this time period (Figures 3B,E,H,K). Deacetylation was found to be more active than acetylation during development (Figures 3B,H vs. E,K). Acetylation was similar between distal and proximal nerve stumps during injury and regeneration (Figures 3E,K), but deacetylation was more active distally than proximal (Figures 3B,H). Based on the number and proportion of genes in each module, we found that regardless of acetylation or deacetylation, module 1 had the largest proportion (Figures 3C,F,I,L). Our results reveal that the genes in module 1 are decisive, that is, most of acetylation regulators have the same changing trend during development and regeneration and the opposite trend during injury, which also implies that protein acetylation is involved in the above three processes.

### Analysis of Pan-Acetylation During Sciatic Nerve Development, Injury, and Regeneration

In addition to expression analysis of acetylation regulators, we also tested changes in overall acetylation levels of the sciatic nerve during development, injury, and regeneration using pan-acetylation antibodies. Total acetylation levels in nerve tissue increased substantially from early development (1 day) to adulthood (6 months), which were approximately threefold higher at 6 months than at 1 day (Figure 4A). Subsequently, we constructed a mouse sciatic nerve clamp model, and Western blot assay was done at 1, 7, and 14 days to analyze the level of pan-acetylation. As expected, the total acetylation level during sciatic nerve injury (N vs. 1d) were ∼2.5-fold down-regulated as opposed to that during development (Figure 4B). During post-injury regeneration (7d vs. 14d), the total acetylation level in sciatic nerve tissue was approximately 1.6-fold up-regulated, which was consistent with that during development but opposite to that during injury (Figure 4C). Our results suggest that changes in overall acetylation during sciatic nerve development, injury, and regeneration are consistent with the regularity of acetylation regulators and may imply that the upregulation of acetylation during development is primarily driven by downregulated deacetylases and upregulated acetyltransferase.

### Key Acetylation Regulators Associated With Cell Cycle-Related Pathways

We sought to find which regulators might act as major genes associated with development, injury, and regeneration. The Venny tool was used to intersect differentially expressed genes during development, injury, and regeneration. Ultimately, Hdac1/2, Kat2b, and Esco2 emerged as differentially expressed genes at any part of any process (Figures 5A–D). Upon examination, Hdac1/2 was down-regulated and Kat2b was up-regulated during development, consistent with the trend of increased pan-acetylation during development.

**FIGURE 6:**
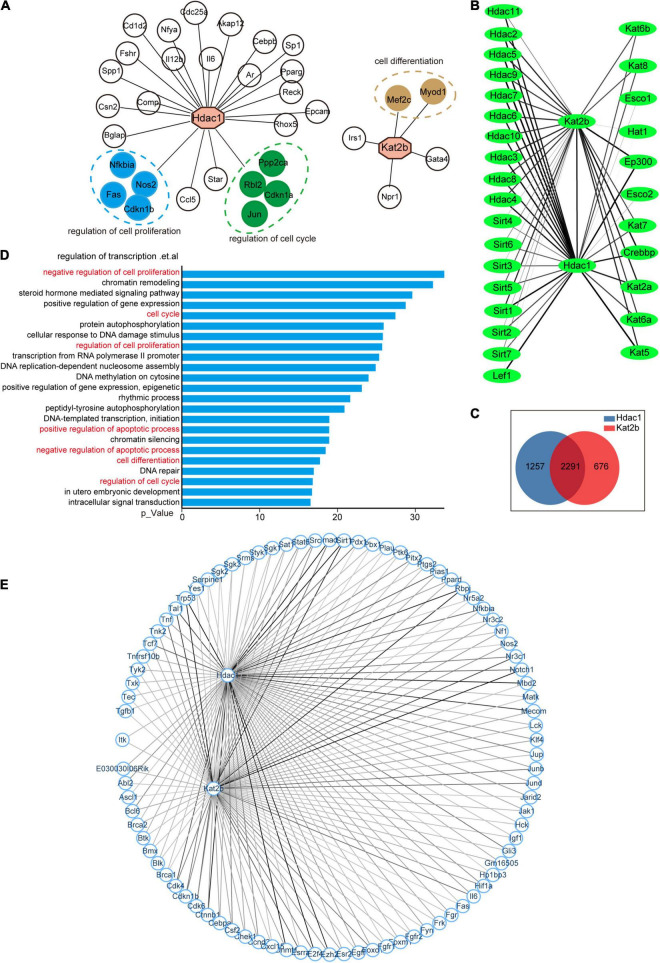
The cell cycle pathway is downstream of Hdac1 and Kat2b. **(A)** Hdac1 and Kat2b act as downstream molecules regulated by transcription factors. Green color indicates that the gene can be clustered in the regulation of cell proliferation pathway, blue color indicates that the gene can be clustered in the regulation of cell cycle pathway, and brown color indicates that the gene can be clustered in the cell differentiation pathway. **(B)** Interaction of Hdac1 and Kat2b with other acetylation regulators. The thickness of a line indicates the intensity of the interaction, and the thicker line indicates the stronger interaction. **(C)** Intersection of molecules that interact with both Hdac1 and Kat2b. **(D)** GO analysis of intersected genes. Pathways related to the cell cycle are marked in red. **(E)** Interaction of Hdac1 and Kat2b with proteins related to the regulation of cell proliferation pathway. The thickness of a line indicates the intensity of the interaction, and the thicker line indicates the stronger interaction.

To uncover the mechanisms underlying key acetylation regulators associated with sciatic nerve development, injury, and regeneration, we analyzed their potential downstream molecules represented by Hdac1 and Kat2b. Acetyltransferases and deacetylases act by regulating both histone acetylation and non-histone acetylation (Marmorstein and Zhou, 2014; Sabari et al., 2017). Therefore, we analyzed the regulatory molecules of Hdac1 and Kat2b from the perspectives of transcriptional activation and protein interaction. A total of 28 and 5 TF-target interaction pairs for Hdac1 and Kat2b were identified respectively using the Transcriptional Regulatory Relationships Unraveled by Sentence-based Text Mining (TRRUST). As transcription factors, the downstream molecules of Hdac1 and Kat2b did not overlap. Functional analysis of Hdac1-targeted molecules indicated that they could be clustered for the regulation of cell proliferation and cell cycle. In contrast, Kat2b-targeted molecules could be aggregated in the cell differentiation pathway (Figure 6A). These pathways were all related to cell cycle. We also used STRING to obtain 3,548 protein–protein interaction pairs for Hdac1 and 2,967 for Kat2b. We found that these two proteins had a general interaction with other acetylation regulators, suggesting that a complex regulatory relationship, co-regulated by multiple enzymes, exists in the intracellular acetylation. Hdac1 showed stronger interaction ability and regulated more molecules than Kat2b (Figure 6B). Unlike transcription factor activity, most of the downstream molecules of Hdac1 and Kat2b were overlapped for protein interaction, suggesting that Hdac1 and Kat2b may work by regulating the same molecules (Figure 6C). GO analysis of these 2,291 molecules indicated that, unsurprisingly, some of the most prominent pathways were mostly clustered in the transcription regulation pathway regulated by histone acetylation. However, for non-histone regulation, most of these molecules can be aggregated in cell cycle-related pathways such as regulation of cell proliferation, cell cycle, cell apoptosis, and cell differentiation (Figure 6D). We focused on the interaction between the two proteins and proteins related to the cell proliferation regulation pathway. The interaction of Hdac1 with this pathway was slightly stronger than that of Kat2b. Among the proteins related to the cell proliferation regulation pathway, Dmnt1, Trp53, Smad4, Mbd2, E2f4, Ctnnb1 interacted more strongly with Hdac1, and Notch1, Trp53, Rbpj, Sirt1, Tal1, Nr3c1 interacted more strongly with Kat2b (Figure 6E). Importantly, these molecules have been clearly reported to have acetylation modifications that affect their own functions (Huang et al., 2014; Kong et al., 2020; Jia et al., 2021). Notch1, Cdk4, Sirt1, and Nr3c1 are confirmed to be closely related to peripheral nerve regeneration or SCs physiological functions (Woodhoo et al., 2009; Viader et al., 2011; Lerch et al., 2017; Romeo-Guitart et al., 2019).

**FIGURE 7:**
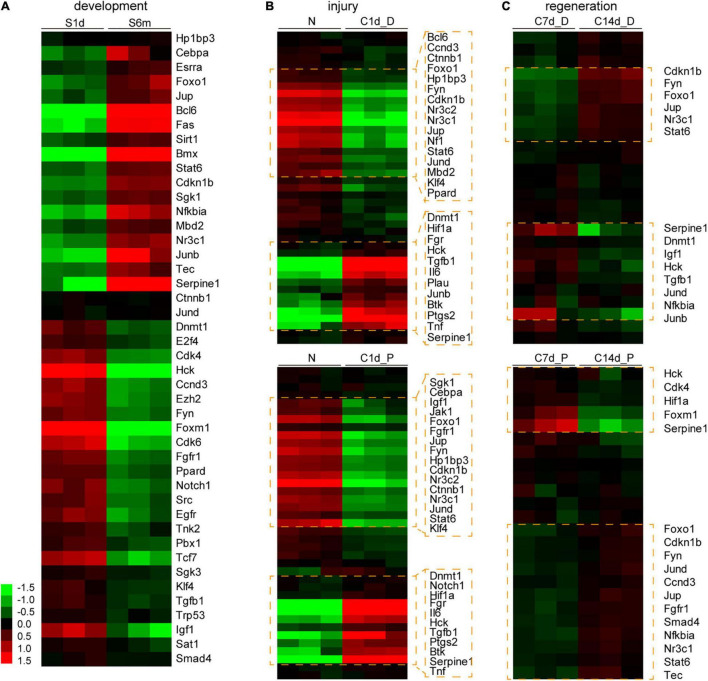
Expression analysis of the downstream genes of Hdac1 and Kat2b during development, injury, and regeneration. Analysis of expression changes of downstream genes of Hdac1 and Kat2b enriched in cell proliferation pathways (shown in Figure 6E) during development **(A)**, injury **(B)**, and regeneration **(C)**. Gene expression data were log transformed, the darker the red, the higher the expression level, and the darker the green, the lower the expression level. Clusters and genes with significant differential expression are marked with orange boxes.

Changes in the expression of downstream molecules shared by Hdac1 and Kat2b in the cell proliferation pathway were analyzed during development, injury, and regeneration. These target genes changed most dramatically during development, followed by injury and milder regeneration (Figures 7A–C). Regardless of injury or regeneration, differentially expressed targets are similar in proximal and distal nerve stumps, consistent with the expression signature of acetylation regulators (Figures 3B,E,H,K, 7B,C). Notably, IL6 is significantly differentially expressed during the injury, suggesting that acetylation may be one of the mechanisms of inflammatory activation after peripheral nerve injury (Figure 7B). The expression changes of most target genes during development, injury, and regeneration are consistent with “development and regeneration are consistent and opposite to injury,” among which representative targets are Foxo1, Cdkn1b, Nr3c1, Jup, Stat6, Jund, and Hck.

**FIGURE 8:**
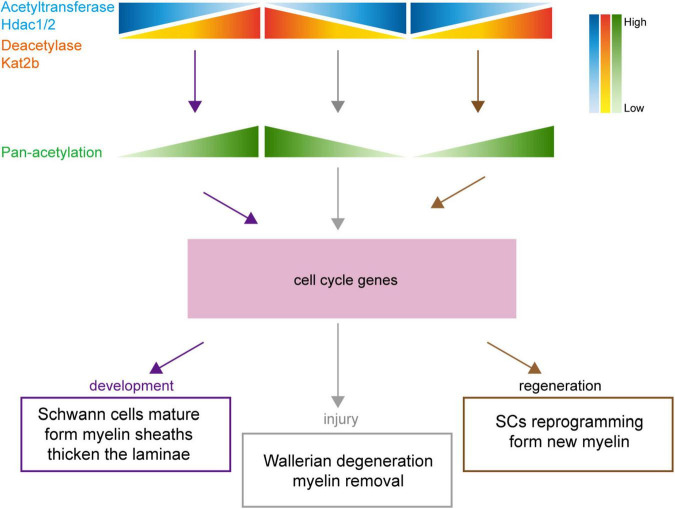
Schematic diagram of the association of protein acetylation with sciatic nerve development, injury, and regeneration. Under the regulation of numerous acetylation regulators, such as Hdac1/2 and Kat2b, changes in protein acetylation link sciatic nerve development, injury, and regeneration. During development, the total acetylation level is increased, driven by Hdac1/2 downregulation and Kat2b upregulation. During injury, the total acetylation level is decreased, driven by Hdac1/2 upregulation and Kat2b downregulation. During regeneration, the total acetylation level is increased, driven by Hdac1/2 downregulation and Kat2b upregulation. The regular changes in acetylation may affect cell cycle-related pathways, resulting in similar physiological changes such as myelination and lamellar thickening during sciatic nerve development and regeneration and inverse physiological changes such as myelin degradation during injury.

Our data suggest that key acetylation regulators such as Hdac1 and Kat2b may mediate morphological changes that are similar or opposite during sciatic nerve development, injury, and regeneration through regulating the acetylation levels of cell cycle pathway-related members (Figure 8).

## Discussion

In this study, we constructed a transcriptome database to systematically analyze the expression changes of all acetylation regulators during sciatic nerve development, injury, and regeneration, and found that their expression changes followed the rule that “the target gene shows the same changes in the development and regeneration process but an opposite trend in the injury process.” Experimental verification indicated that peripheral nerve development and regeneration were processes of increased pan-acetylation while injury was a process of decreased pan-acetylation, verifying the variation rule of the expression of acetylation regulators. Moreover, we predicted the molecules that have regulatory relationships with two key acetylation regulators, Hdac1 and Kat2b, and found numerous overlapped molecules mainly related to cell cycle-related pathways. Therefore, our data implicate that protein acetylation may be a bridge between peripheral nerve development, injury, and regeneration by reveal the regularity of acetylation regulators during peripheral nerve development, injury, and regeneration.

A large amount of omics data have facilitated the physiological and pathological research of peripheral nerves and uncovered many new discoveries in this field. Several studies have focused on gene expression changes during nerve degeneration and regeneration following sciatic nerve injury in rats and provided two databases: RNA-seq and microarray (Wang et al., 2012, 2021b; Yi et al., 2015, 2017). However, the sciatic nerve transcriptome data of different age groups are also obtained from rats (Liu et al., 2018; Djuanda et al., 2021). There is still a lack of mouse-derived databases for the sciatic nerve, possibly due to the difficulty in sciatic nerve sample harvesting. The mouse transcriptome database from the present study is a supplement to the previous databases. Moreover, a total of 11 observational time points from pre-birth (embryonic day 20) until postnatal 12 months were set in this study, which are more intensive than the previous data. For example, a study by Liu et al. (2018) provided relevant data from 1-week-old and 12-month-old healthy Sprague-Dawley rats; and Djuanda et al. (2021) selected 1- and 24-month-old Sprague-Dawley rats as experimental animals. Therefore, although we only focus on the expression changes of acetylation regulators, our study still provides two unprecedented high-quality databases, with a huge amount of data waiting to be mined.

Although development and regeneration are inextricably linked, there has been always a debate on whether regeneration is a recapitulation of development or a unique phenomenon independent of development. When an injury occurs, tissues and organs in the body experience a secondary development – regeneration by mobilizing the expression of genes that are originally activated only during development (Lu et al., 2020; Wang et al., 2020). The other view is that development and regeneration are fundamentally different processes only with some overlapped cellular and genetic mechanisms (Bideau et al., 2021). Our data reflect the first view that regeneration is a recapitulation of development and highlight that protein acetylation may be a mechanism for the association between development and regeneration. In addition, our data also reveal that after sciatic nerve injury, the level of acetylation in adult mice rapidly returns to the level at the beginning of development and re-experiences similar developmental changes upon regeneration. Protein acetylation changes match the phenomena below: after peripheral nerve injury, myelinated SCs transdifferentiate into proliferating progenitor-like SCs and further re-differentiate into myelinated SCs (Stierli et al., 2018).

After nerve injury, SCs from both distal and proximal nerve stumps undergo dedifferentiation in the early stage (Jessen and Mirsky, 2016). However, there are different physiological changes in distal and proximal nerve stumps. Axon regeneration initially starts in the proximal nerve stump, and to create an environment suitable for subsequent axon regeneration, Wallerian degeneration occurs in the distal nerve stump, characterized by myelin sheath decomposition, cytoskeleton degradation, and SCs proliferation, causing SCs extending longitudinally to form the bands of Büngner (Min et al., 2021). A recent study found that there were different gene expression profiles in proximal and distal nerve stumps after sciatic nerve injury in rats, and several pathways related to nerve regeneration, such as inflammation and metabolism, were enriched in distal and proximal nerve stumps to different extents (Lu et al., 2022). During nerve injury and regeneration, acetylation-related activities, especially deacetylation, in the distal nerve stump is more active than those in the proximal nerve stump. This finding is consistent with the physiological changes of proximal and distal nerve stumps during injury and regeneration, suggesting that acetylation of more proteins is required for Wallerian degeneration. Regardless of the development process or the comparison between the proximal and distal nerve stumps, changes in deacetylase expression are stronger than those in acetyltransferase expression. This indicates that deacetylase-mediated deacetylation is involved more in sciatic nerve development, injury, and regeneration, which is also reflected by the stronger interaction between Hdac1 and its downstream molecules than that between Kat2b and its downstream molecules.

Histone deacetylase 1 is a widely studied regulator of peripheral nerve injury and regeneration, but little about Kat2b. Our study suggests that Kat2b may cooperate with Hdac1 to participate in peripheral nerve injury and regeneration through common targets. Previous studies have confirmed that Hdac1 is up-regulated in the early stage and down-regulated in the late stage of peripheral nerve injury, which is consistent with the sequencing results in this study (Brugger et al., 2017; Dzreyan et al., 2021). The most common function of Hdac1 is to deacetylate histones, which functions through epigenetic (Jacob et al., 2011; Wu et al., 2016; Brugger et al., 2017). Recent studies suggested that Hdac1 can also regulate SC myelination by deacetylating non-histone proteins such as eEF1A1 and NF-κB (Chen et al., 2011; Duman et al., 2020). Our data suggest that Hdac1 or Kat2b may also play a role in peripheral nerve injury and regeneration by regulating the acetylation of non-histone proteins such as Foxo1, Cdkn1b, Nr3c1, Jup, Stat6, Jund, and Hck. Wallerian degeneration is accompanied by activation of inflammatory factors such as Il6 (Camara-Lemarroy et al., 2010). Our data suggest Il6 as targets of Hdac1 and Kat2b, implying that protein acetylation may be one of the mechanisms of inflammatory activation in the early stages of injury. This is also suggested by the fact that the use of the histone deacetylase inhibitor SAHA reduces Hdac1/2 expression and alleviates inflammation (He et al., 2020).

Protein acetylation is directly related to the cell cycle. SMC3 acetylation catalyzed by Esco1/2 shows evidence of directly affecting the cell cycle by regulating sister chromatid cohesion (Rolef Ben-Shahar et al., 2008). Hdac8 has been subsequently found to directly influence cell cycle by catalyzing SMC3 deacetylation (Deardorff et al., 2012). In addition, acetylation also regulates several other major cell cycle regulators, including P53, protein kinases BUBR1, Aurora kinase A/B, cyclin-dependent kinase 1 (CDK1), CDK2, and PLK4 (Choudhary et al., 2009; Narita et al., 2019). Development, injury, and regeneration of peripheral nerves are both active processes of cell proliferation and differentiation. So, it well understood that protein acetylation is related to the development, injury, and regeneration of peripheral nerves by affecting the cell cycle. Although protein acetylation occurs widely in mammalian cells (Choudhary et al., 2009), it is unclear about in which molecules acetylation is altered during sciatic nerve development, injury, and regeneration and how these molecules influence the cell cycle and the progression of development, injury, and regeneration.

The sciatic nerve is a complex tissue containing neuronal axons, SCs, fibroblasts, vascular cells, and macrophages. Since SCs are the cells with the highest proportion in peripheral nerves, the changes in protein acetylation and acetylation regulator expression described in this study are most likely provided by SCs. Recent evidence reveals that the cells in the nervous system are widely heterogeneous, that is, even cells of the same type may have multiple subtypes that perform different functions, further characterizing the structural complexity and functional diversity of the nervous system (Sun et al., 2021; Zhang et al., 2021). Therefore, the major changes in protein acetylation may be originated from one or several cell subtypes that are associated with development or regeneration. Future explorations are needed on the regularity of acetylation regulators of single cell types during development, injury, and regeneration.

## Data Availability Statement

The original contributions presented in the study are included in the article/Supplementary Material, further inquiries can be directed to the corresponding authors.

## Ethics Statement

All animal experiments were approved by the Animal Ethics Committee of Nantong University and followed the Chinese Guidelines for the Care and Use of Laboratory Animals. The tester was qualified to have the Jiangsu Provincial Laboratory Animal Practitioner Post Certificate with a certificate no. 220212305.

## Author Contributions

JS, MS, and FD designed the study and wrote the manuscript. JS, YJ, MM, YC, QZ, SZ, and MS performed the experiments. JS and YJ collected and assembled data. JS, MS, and QL performed data analysis and revised the manuscript. All authors read the manuscript and agreed to publish it.

## Conflict of Interest

The authors declare that the research was conducted in the absence of any commercial or financial relationships that could be construed as a potential conflict of interest.

## Publisher’s Note

All claims expressed in this article are solely those of the authors and do not necessarily represent those of their affiliated organizations, or those of the publisher, the editors and the reviewers. Any product that may be evaluated in this article, or claim that may be made by its manufacturer, is not guaranteed or endorsed by the publisher.

## Footnotes

1
http://imagej.nih.gov/ij

2
https://cytoscape.org/
